# 

**Published:** 1847-01-01

**Authors:** 


### CONTENTS

OF THE

### MEDICO-CHIRURGICAL REVIEW,

### JANUARY 1, 1847.

### REVIEWS.

I.

I. The Works of William Hewson, F.R.S. Edited by GEORGE GULLIVER, F.R.S.
II. The Blood-Corpuscle considered in its different Phases of Development in the Animal Series. By J. WHARTON JONES, F.R.S. .. .. .. .. .. .. 1

1. Biography of Hewson .. .. .. .. .. .. .. .. .. .. 3

2. Estimate of Hewson's Labours .. .. .. .. .. .. .. .. 5

3. Condition of the Blood in Inflammation .. .. .. .. .. .. 7

4. Influence of System on Coagulation .. .. .. .. .. .. .. 9

5. Formation of the Red Corpuscles .. .. .. .. .. .. .. .. 11

6. Blood in the Embryo .. .. .. .. .. .. .. .. .. .. 15

7. Development of the Red Corpuscles .. .. .. .. .. .. .. 17

8. On the Villi of Fishes and Reptiles .. .. .. .. .. .. .. 19

II.

I. Health of Towns' Association. Report of the Committee to the Members of the Association on Lord Lincoln's Sewerage, Drainage, &c. of Towns Bill
II. Unhealthiness of Towns, its Causes and Remedies. By Viscount EBRINGTON, M.P. .. .. .. .. .. .. .. .. .. .. .. ..
III. On the Health of Towns, as influenced by defective Cleansing and Draining, and on the Application of the Refuse of Towns to Agricultural Purposes. By WILLIAM A. GUY, M.B. Cantab. &c ... .. .. .. .. .. .. ..
IV. The Liverpool Health of Towns Advocate. Edited by JOHN SUTHERLAND, M.D. &c. .. .. .. .. .. .. .. .. .. .. .. .. .. ..
V. The Improvement of Manchester. A Report setting forth a Plan proposed by the Towns' Improvement Company, &c. .. .. .. .. .. .. .. 21

1. Defects of Lord Lincoln's Bill.. .. .. .. .. .. .. .. .. 22

2. Duties of the Officer of Health .. .. .. .. .. .. .. .. 25

3. Conclusions respecting the Sanitary Bill .. .. .. .. .. .. 27

4. Proceedings of the Liverpool Association .. .. .. .. .. .. 29

5. Lord Ebrington's Lecture .. .. .. .. .. .. .. .. .. 30

III.

Lectures on Subjects connected with Clinical Medicine, comprising Diseases of the Heart. By P. M. LATHAM, M.D. &c... .. .. .. .. .. .. .. .. 33

1. Secondary Endocarditis and Pericarditis .. .. .. .. .. .. 35

2. Treatment of Endocarditis and Pericarditis .. .. .. .. .. .. 37

3. Adhesion of the Pericardial Surfaces, &c. .. .. .. .. .. .. 39

4. Certain Peculiarities in grave Organic Lesions of the Heart.. .. .. 41

5. Injuries of the Valves from violent Muscular Efforts .. .. .. .. 43

6. Hypertrophy resulting from various Causes .. .. .. .. .. .. 45

7. Diagnosis and Treatment of Hypertrophy .. .. .. .. .. .. 47

IV.

I. Manuel des Accouchments et des Maladies des Femmes Grosses et Accouchées contenant les Soins a donner aux Nouveaux-nes. Par J. JACQUEMIER, M.D.
II. Recherches et Considerations sur la Constitution et les Fonctions du Col de l'Uterus. Par C. NEGRIER.. .. .. .. .. .. .. .. .. .. 49

1. Negrier on the Neck of the Womb.. .. .. .. .. .. .. .. 51
2. Jacquemier on the Disorders of Menstruation .. .. .. .. .. 53

3. Jacquemier on Hydrorrhœa .. .. .. .. .. .. .. .. .. .. .. 54

4. Jacquemier on the Ergot of Rye .. .. .. .. .. .. .. .. .. .. 57

5. Negrier on Placental Presentations.. .. .. .. .. .. .. .. .. 59

6. Negrier on Uterine Hæmorrhage .. .. .. .. .. .. .. .. .. 61

7. Jacquemier on Embryotomy .. .. .. . .. .. .. .. .. .. .. 65

V.

Clinical Collections and Observations in Surgery. By W. P. Ormerod.. .. .. 67

1. Fracture of the Thigh, Leg, &c. .. .. .. .. .. .. .. .. .. .. 68

2. Discharge of Serous Fluid from the Ear in Fracture of the Skull .. 70

3. Treatment of enlarged Subcutaneous Bursæ.. .. .. .. .. .. .. 70

4. Amputation .. .. .. .. .. .. .. .. .. .. .. .. .. .. .. 71

5. Treatment of Syphilis .. .. .. .. .. .. .. .. .. .. .. .. .. 73

6. Mercury and Iodine in Syphilis .. .. .. .. .. .. .. .. .. 74

VI.

Recueil de Memoires de Medecine, de Chirurgie, et de Pharmacie Militaires.. .. 78
1. On the Climate and Diseases of Algeria. By Dr. Casimir Broussais.. 79
2. Researches on the Condition of the Blood in the Endemic Diseases of Algeria. By MM. Leonard and Foley .. .. .. .. .. .. .. .. 85
3. Cases of Wounds and Diseases of the Brain, which tend to prove that the Faculty of Language is placed in the Anterior Lobes of that Organ. By M. Bonnafont .. .. .. .. .. .. .. .. .. .. .. .. .. .. 86
4. On Foreign Bodies in the Ear. By M. Latour .. .. .. .. . .. 87

VII.

Guy's Hospital Reports. Second Series. Edited by Drs. Barlow and Birkett, and Messrs. Cock and Poland. Vol. IV... .. .. .. .. .. . .. .. 90
1. On the Fallacies attending Physical Diagnosis in Diseases of the Chest. By Thomas Addison, M.D., &c. .. .. .. .. .. .. .. .. .. .. .. 90
2. Examples of Ptosis, with illustrative Remarks. By John F. France.. 98
3. Reports of the Clinical Society, from January 1845 to March 1846. By Alfred Poland .. .. .. .. .. .. .. .. .. .. .. .. .. .. 100
4. Clinical Report of Cases admitted into the Petersham Ward. By John C. W. Lever, M.D. .. .. .. .. .. .. .. .. .. .. .. .. .. 104
5. On the Physiology of Cells, with the View to elucidate the Laws regulating the Structure and Function of Glands. By Thomas Williams, M.D. .. .. .. .. .. .. .. .. .. .. .. .. .. .. .. .. .. 106
6. Case of supposed Spontaneous Perforation of the Stomach terminating successfully. By Dr. Hughes. .. .. .. .. .. .. .. .. .. .. 107
7. Ulcer of the Stomach, leading to Perforation and Death in 19 hours. By Mr. Ray and Mr. Hilton.. .. .. .. .. .. .. .. .. .. .. 107
8. Appearances in the Stomach after Death. By Mr. Wilkinson King .. 109
9. Dr. Hughes on Chorea .. .. .. .. .. .. .. .. .. .. .. .. .. 109
10. Cases and Observations in Medical Jurisprudence. By Alfred Taylor, F.R.S. .. .. .. .. .. .. .. .. .. .. .. .. .. .. .. .. .. 112

VIII.

I. Conseils aux Mères sur l'Allaitement et sur la Maniere d'Elever les Enfans Nouveau-nés. Par Al. Donne, D.M., &c. .. .. .. .. .. .. .. ..

II. Dr. Underwood's Treatise on Children, &c. By Henry Davies, M.D., &c. } 117

1. On Suckling .. .. .. .. .. .. .. .. .. .. . .. .. .. 118

2. Wet-nurses—Composition of Milk .. .. .. .. .. .. .. .. 119

3. Morbid Changes of the Milk .. .. .. .. .. .. .. .. .. .. .. 121

4. Country-air and Milk Regimen .. .. .. .. .. .. .. .. .. .. 122

5. Materia Medica for Children .. .. .. .. .. .. .. .. .. .. .. 124

6. Laryngismus Stridulus—Worms .. .. .. .. .. .. .. .. .. .. 129

7. Treatment of Pertussis and Porrigo .. .. .. .. .. .. .. .. .. 131

IX.

On the Pathology and Treatment of Scrofula. By Robt. Mortimer Glover, M.D., &c. . .. .. .. .. .. .. .. .. .. .. .. .. .. .. .. .. 132

1. Identity of Scrofulous and Tuberculous Matter .. .. .. .. .. .. 132

2. Essential Nature of Scrofula .. .. .. .. .. .. .. .. .. .. .. 135

3. Mode of Action of Cod-liver Oil and Iodine .. .. .. .. .. .. .. 137

X.

I. Animal Chemistry, or Chemistry in its Applications to Physiology and Pathology. By Baron Liebig. Edited by W. Gregory, M.D., F.R.S.E. ..
II. Experimental Researches on the Food of Animals and the Fattening of Cattle, with Remarks on the Food of Man. By Robert Dundas Thomson, M.D. } 138

1. Origin and Uses of the Bile .. .. .. .. .. .. .. .. .. 139

2. The Respiratory Elements of Food.. .. .. .. .. .. .. .. 140

3. The Intestinal Canal an Organ of Secretion .. .. .. .. .. .. 142

4. Influence of Variety of Food .. .. .. .. .. .. .. .. .. 143

5. On the Food of Children.. .. .. .. .. .. .. .. .. .. 145

6. Necessity of practised Observation.. .. .. .. .. .. .. .. 147

7. The Chemical and Parasitic Theories of Contagion .. .. .. .. 148

XI.

Practical Observations and Suggestions in Medicine. By Marshall Hall, M.D. F.R.S. L. & E., &c. .. .. .. .. .. .. .. .. .. .. .. .. 151

1. Seat of the Passions and Sense of Pain .. .. .. .. .. .. .. 153

2. Theory as to the Cause of Sleep .. .. .. .. .. .. .. .. 154

3. Experiments on Living Animals reprobated .. .. .. .. .. .. 157

4. Remarks on some Puerperal Diseases .. .. .. .. .. .. .. 159

5. Flourens' Experiments on Birds .. .. .. .. .. .. .. .. 160

6. Plan for the Institution of Bloodletting.. .. .. .. .. .. .. 162

7. The Author's Merits and Failings .. .. .. .. .. .. .. .. 164

XII.

Seventh Annnal Report of the Registrar-General of Births, Deaths, and Marriages, in England. (Abstracts of the two years, 1843, 1844) .. .. .. .. .. 166

XIII.

I. The Moral Aspects of Medical Life; consisting of the "Akesios" of Professor H. F. K. Marx. Translated from the German, &c., by James Mackness, M.D., &c. &c... .. .. .. .. .. .. .. .. .. .. .. ..
II. Life of George Cheyne, M.D., with Extracts from his Works and Correspondence .. .. .. .. .. .. .. .. .. .. .. .. .. .. } 171

1. Life and Character of Boerhaave .. .. .. .. .. .. .. .. 172

2. Life and Character of Mead .. .. .. .. .. .. .. .. .. 179

3. Life and Character of Cheyne .. .. .. .. .. .. .. .. 183

4. Life and Character of Hallé .. .. .. .. .. .. .. .. .. 191

XIV.

I. Practical Remarks on Near-Sight and Impaired Vision. By William White Cooper.. .. .. .. .. .. .. .. .. .. .. .. ..
II. A Manual of the Diseases of the Eye. By S. Littel, Jun. M.D. .. ..
III. Annales d'Oculistique. Tom. XIII.—XVI. .. .. .. .. .. .. } 196

1. On the Use of Spectacles .. .. .. .... .. .. .. .. .. 197

2. Management of Presbyopia or Far-sightedness .. .. .. .. .. 199

3. On Amblyopia resulting from Presbyopia .. .. .. .. .. .. 201

4. Consequences of Presbyopia .. .. .. .. .. .. .. .. .. 203

5. Preservation of the Eyes—Myopia .. .. .. .. .. .. .. 205

6. Impaired Vision—Artificial Light .. .. .. .. .. .. .. .. 207

XV.

On Diseases of the Skin. By Erasmus Wilson, F.R.S., &c. .. .. .. .. .. 209
1. Structural Anatomy of Hair.. .. .. .. .. .. .. .. .. .. 210
2. Pathology of Lichen and Prurigo .. .. .. .. .. .. .. .. 212
3. Trichosis Furfuracea, or Ring-worm.. .... .. .. .. .. .. 214

XVI.

Medico-Chirurgical Transactions:— .. .. .. .. .. .. .. .. .. .. 216
1. On the Minute Anatomy and Pathology of Bright's Disease of the Kidney, &c. &c. By George Johnson, M.D... .. .. .. .. .. 216
2. Account of a Case of Congenital Deficiency of one Kidney, with Granular Degeneration of the existing one. By George Busk, Surgeon, &c. 222
3. On the Intimate Structure of the Human Kidney, &c. By Joseph Toynbee, F.R.S. &c... .. .. .. .. .. .. .. .. .. .. 225

4. History of a Case of Ligature of the Left Subclavian Artery between the Scaleni Muscles, &c. By J. C. Warren, M.D. &c. .. .. .. .. .. 228
5. Two Cases of Disease of the Brain, following the Application of a Ligature of the Carotid Artery. By John P. Vincent, Esq. &c. .. .. 230
6. Case of Punctured Wound and Ligature of the Posterior Tibial Artery in the Upper Third of its Course. By James Moncrieff Arnott, F.R.S. &c. .. .. .. .. .. .. .. .. .. .. .. .. .. .. .. .. .. 232

XVII.

A Manual of Materia Medica and Therapeutics, including the Preparations of the Pharmacopœias of London, Edinburgh and Dublin, with many New Medicines. By J. Forbes Royale, M.D., F.R.S. &c. .. .. .. .. .. .. .. .. .. .. .. 234

1. Action and Uses of Medicines .. .. .. .. .. .. .. .. .. .. .. 235

2. Description of the Sulphate of Iron .. .. .. .. .. .. .. .. .. 236

3. The Assafœtida Plant .. .. .. .. .. .. .. .. .. .. .. .. .. 239

4. Account of Bebeerine .. .. .. .. .. .. .. .. .. .. .. .. .. 240

5. Account of Cannabis Indica .. .. .. .. .. .. .. .. .. .. .. .. 241

6. Therapeutics .. .. .. .. .. .. .. .. .. .. .. .. .. .. .. .. 243

### Bibliographical Notices, &c.

Observations on Hydropathy, with an Account of the principal Cold-Water Establishments of Germany. By J. Stevenson Bushnan, M.D. .. .. } 245
A Review of Homœopathy, Allopathy, and Young Physic. By L. M. Lawson, M.D. &c. .. .. .. .. .. .. .. .. .. .. .. .. .. .. .. }
Liebig's Question to Mulder, tested by Morality and Science. By Dr. G. T. Mulder, &c... .. .. .. .. .. .. .. .. .. .. .. .. .. .. .. .. .. 247
Chemistry of the Four Seasons. By Thomas Griffiths .. .. .. .. .. .. .. 248
The Use of the Body in relation to the Mind. By George Moore, M.D. &c... .. 249
The Potatoe Plant, its Uses and Properties, &c. By Alfred Smee, F.R.S. .. .. 251
Notes on the Epidemic Cholera. By R. Hartley Kennedy, M.D. &c. .. .. .. 252
The Microscopic Anatomy of the Human Body in Health and Disease. By Arthur Hill Hassall .. .. .. .. .. .. .. .. .. .. .. .. .. .. .. .. .. 253
The Handbook of Human Anatomy, General, Special, and Topographical. Translated from the German of Von Behr. By John Birkett, Surgeon, &c. .. .. 254
Principles of Human Physiology, &c. By William B. Carpenter, M.D. F.R.S. &c. 254
Transactions of the Medical and Physical Society of Bombay, for the Year 1844.. 255
A Tabular View of the Physical Signs and Diagnosis of the Diseases of the Lungs, &c. By James Turnbull, M.D. &c. .. .. .. .. .. .. .. .. .. .. .. 256

### PERISCOPE.

### Selections from the Foreign Periodicals.

1. On the Retrospective Semiological Characteristics presented by the Nails. By J. Beau, M.D. .. .. .. .. .. .. .. .. .. .. .. .. .. .. .. .. 257

2. Blisters in Confluent Small-Pox .. .. .. .. .. .. .. .. .. .. .. .. .. 258

3. Harden on Isopathia .. .. .. .. .. .. .. .. .. .. .. .. .. .. .. .. 259

4. M. Malgaigne on Simple Inflammation or Pseudo-strangulation of Hernias .. 260

5. On the Influence which Diseases of the Uterus exert in producing a Faulty Position of the Child .. .. .. .. .. .. .. .. .. .. .. .. .. .. 262

6. M. Velpeau on Granulations of the Cervix Uteri .. .. .. .. .. .. .. . 263

7. On the Organic Causes and the Mechanism of the Production of Hysteria .. 264

8. On the Employment of Iodide of Potassium in the Treatment of Syphilis .. 267

9. On the Employment of Diluents in Urethritis .. .. .. .. .. .. .. .. .. 269

10. Fracture of the Cervix Femoris .. .. .. .. .. .. .. .. .. .. .. .. .. 270

11. Treatment of Saturnine Affections .. .. .. .. .. .. .. .. .. .. .. .. 270

12. Lombard on Sudden Death .. .. .. .. .. .. .. .. .. .. .. .. .. .. 272

13. Puerperal Convulsions—Malpraxis .. .. .. .. .. .. .. .. .. .. .. .. 274

14. On the Elevation of Parts in the Treatment of some Surgical Diseases .. .. 275

15. M. Sichel on the Use and Abuse of Mercurial Preparations .. .. .. .. .. 276

16. M. Saucerotte on Latent Pneumonia .. .. .. .. .. .. .. .. .. .. .. . 277

17. Dr. Neucourt on Epidemic Erysipelas .. .. .. .. .. .. .. .. .. .. .. .. 279

18. On the Nature of Insanity, and its Treatment by Irrigation and prolonged Baths. By M. Brie[illegible] .. .. .. .. .. .. 281

